# Development of overcurrent relay based on wavelet transform for fault detection in transmission line

**DOI:** 10.1038/s41598-024-65596-y

**Published:** 2024-06-28

**Authors:** Santipont Ananwattanaporn, Praikanok Lertwanitrot, Atthapol Ngaopitakkul, Chaichan Pothisarn

**Affiliations:** 1 School of Engineering, King Mongkut’s Institute of Technology Ladkrabang, Bangkok, Thailand; 2 Electricity Generating Authority of Thailand, Nonthaburi, Thailand

**Keywords:** Protection system, Wavelet transform, Transmission line, Fault detection, Electrical and electronic engineering, Power distribution

## Abstract

This study proposes a protection relay using a microcontroller to detect and classify faults in transmission lines based on the wavelet transform. An experimental model was constructed from an actual 115 kV transmission system prototype. The current signal was observed based on the fault type, phase, and position. Clark’s transform and the discrete wavelet transform (DWT) were applied to transform signals for analysis. Moreover, the performance of fault detection based on the output signals of Clark’s transform (alpha sequence, beta sequence, and zero sequence current) was compared to the performance of the alternative proposed fault detection method, which is based on the combining factor between alpha and beta sequence current. In addition, the influence of DWT level on fault analysis is also considered and is used to confirm the accuracy of fault detection. Results show that the proposed method is efficient for fault detection and classification. This finding allows the researcher to choose the appropriate analytical method. Moreover, it can also be used as the basis for overcurrent relay algorithm design in the effort to develop more advanced technologies.

## Introduction

Protection systems are highly important in electrical power systems. The best protection system must detect faults quickly and with high accuracy to reduce the damage that may spread. Protecting system performance is essential because it affects the reliability of power systems. Moreover, the best protection system must have high accuracy in identifying faults. Typically, a support maintenance team ensures that affected systems are promptly restored.

A relay is a protection device that responds based on determined conditions. Fault signals are typically input to the relay. There are various types of relay-protection methods. Popular relay-protection types include overcurrent and distance relays^1–5^. The overcurrent relay analyses the current signal by comparing the current magnitude with a predetermined setting. The advantage of using an overcurrent relay is the small amount of data required for the analysis. However, the overcurrent method does not identify the fault location. An additional distance relay can be used to identify the fault location^6,7^. This type of relay uses current and voltage data to calculate the impedance. The impedance ratios can then be converted into transmission line distances.

Conversely, calculating impedance by using current and voltage data is also disadvantageous because the calculated distance has an approximate value and may lead to errors. This has a direct negative effect on the transmission systems. The fault destroys the equipment of the transmission system and increases the possibility of a power outage if the fault cannot be eliminated in time. This resulted in an unstable power system.

To solve the restrictions of traditional relays, several methods were studied and proposed^8–20^ such as the travelling method^8–11^, the Fourier transform^12,13^, and the Wavelet transform^14–16^.

First, when a fault occurs in the transmission line, an impulse wave is generated at the fault location. Due to the impulse energy, the pulse travels to the end of the transmission line. Based on the fault behaviour, the travelling-wave method was applied in the fault analysis to increase the accuracy of fault detection. It uses reflected waves to determine the location of a fault. The time difference and the speed of the waves were the parameters used for the analysis. However, because the travelling-wave method involves complex calculation procedures and the reflected waves have small (hardly detectable) amplitudes, errors may also affect the results.

Based on the aforementioned reasons, the design of a protection system based on the principle of overcurrent relay was presented^17,18^ The advantage of combining both methods is that it only requires current signals. This method is suitable for microcontroller systems. Generalised signal analyses were based on the root-mean-square (RMS). The RMS analyses were conducted in the time domain.

However, due to the fault occurrence, the frequency increased due to the fault energy. Therefore, the benefit of combining the FT with the RMS is less parameter detection and flexibility considerations since the detection data can be analysed in both the time and frequency domains. However, the data used for analysis typically spans at least one period. Therefore, considerable computational power is required to extract a single signal period. Similarly, it was difficult to determine the frequency of the analysis. Moreover, the fault signals are nonstationary. They exhibit different characteristics, depending on the fault. Therefore, the analysis of the fault signal must consider the frequencies of at least two bands close to the frequency band of the fault. Therefore, the Wavelet Transform (WT) was proposed to solve these problems.

The Wavelet method has been extensively used in electrical system analyses. The Wavelet method was developed to analyse the time-domain signals and isolate the components of the signal at different frequencies. Numerous prior studies^19,20^ used the discrete Wavelet transform (DWT) to analyse fault signals. When faults occur, the magnitude of the fault signal changes, and the phase causes signal distortion. The advantage of DWT is that it can detect faults quickly, thus necessitating a small amount of data for analysis.

Since the actual electrical system is complex, experimentally modelling it is difficult. Therefore, many previous studies have investigated the faults generated in complex systems using computer simulations. Correspondingly, various commonly used software programmes are available that commonly use (among others) ATP/EMTP^21,22^, PSCAD^23,24^, and MATLAB^25^. The results of these studies will help researchers better understand electrical systems. They can be used to solve and predict fault situations.

Based on the literature reviews^1–25^, the advantages and disadvantages of traditional methods can be divided as shown in Table 1.

**Table 1:** Liturature review on the traditional fault detection methods.

Method	Advantages	Disadvantages
Config of traditional relay^1–5^	Currently use in high voltage power system of Thailand Reasonable price Easy to maintain	Only the fault detection function can be performed. Unable to determine fault location The accuracy of fault detection function deserves further development
Travelling method^8–11^	Can be used to specify the fault location in transmission line	The signal used to detect traveling waves must be properly transformed first For example; Current signal generating from transmission system was extracted to wavelet signal before analysis by traveling method. The reason was the original signal has noise and high frequency effect The accuracy of the traveling wave is reduced when used in more complex electrical systems. It suitable for use in single end terminals
Fourier transform^12,13^	Can be used to analyze faults in high voltage power system Processing time and accuracy are good	Usage conditions are limited by having to choose to analyze only frequencies or magnitudes on one-time domain
Wavelet transform^14–16^	Developed further from the Fourier transform. Fourier constraint has been solved which parameter of frequencies and magnitudes can be extracted on one-time domain It is widely used in research related to power systems and the performance is satisfactory	Not suitable for analyzing large power systems Most analyzes are based on simulations only. The effectiveness of this in practice is still questionable

Therefore, t is concluded that a high-frequency impulse occurs in the fault position and travels to the end of the line later. The Wavelet Transform method can analyse fault signals in both the frequency and time domains. The PSCAD program can be used to simulate faults in power systems accurately. In addition, to address the incompleteness of traditional studies, this study investigated the faults that occurred in Thailand’s real-world power system. The experiment was performed using both simulations and real-world experiments. First, this study focuses on the characteristics of fault signals generated from the transmission system. All fault types (single-line (SL), line-to-line (LL), double-line-to-ground (DLG), and three-phase (3P) faults) were observed when the phase and location of the fault varied. Current and voltage were considered. However, the focus of this study was on an overcurrent relay based on current data.

In addition, the efficiency of the fault analyses conducted in this study can be improved using methods such as Clark’s transform and WT. Moreover, an alternative factor (positive sequence current), which combines the output of Clark’s transform (alpha sequence current and beta sequence current), is also presented in this study. The trustworthiness of this research was achieved using the fault signal generated in the experimental model set up at the King Mongkut's Institute of Technology Ladkrabang (KMITL) laboratory. The prototype of the experimental model was based on an actual 115 kV transmission system in Thailand. The performance of the proposed method was verified in terms of accuracy and detection time.

The results show that the optimal setting conditions (such as a suitable analysis method and sampling rate) result in high accuracy and short processing times. These findings are beneficial and can be used as a basis for designing fault-detection transmission system algorithms. This can simultaneously lead to performance improvements in relay devices.

The major contributions of this study are as follows:

- An actual 115 kV transmission system was used to prototype the studied system.
- An experimental model was established in the KMITL laboratory to verify the performance of the proposed method.
- The performances of different levels of discrete wavelet transform (DWT) were compared. The results can be used as guidelines for selecting appropriate methods.

Moreover, this study revealed two novel issues that surpass traditional methods: (1) applying the DWT and Clark’s transform in signal analysis, and (2) analysing faults using positive sequence current. This method allows errors in the original method to be fixed. This study comprises four sections. Section "Experimental" describes the experiments conducted. The fault detection and classification processes are presented in Section "Use of WT for fault detection and classification". Section "Discussion and analysis" presents a discussion and analysis, and the last section concludes the study.

## Experimental

**Figure 1:**
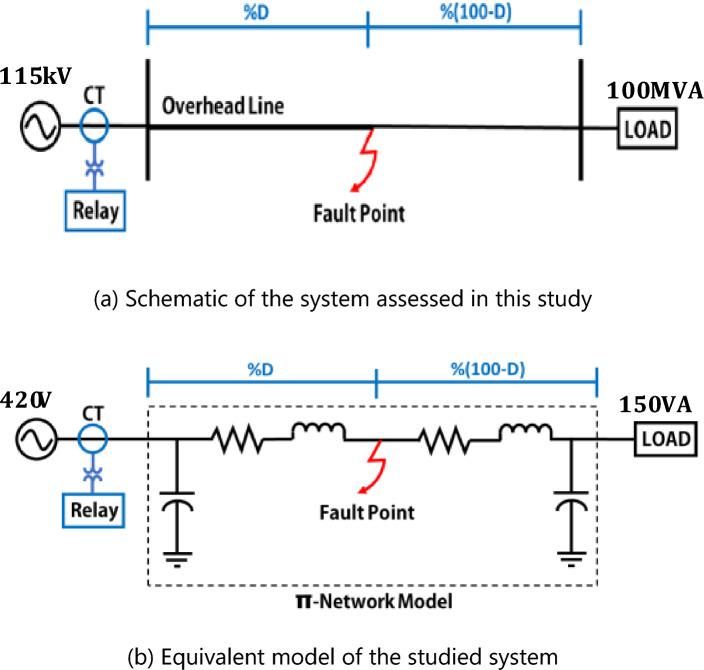
Schematic of the electric system assessed in this study and its equivalent model.

The experiments conducted in this study are described in this section. An experimental model was established at the KMITL laboratory. The experimental model was created as an equivalent system to the actual 115 kV transmission system. The prototype system is displayed in Fig. 1a, showing a 115 kV substation connected to a load of 100 MVA and a 40 km transmission line between the substation and the load. A current transformer (CT) and relay were connected to the substation to detect the current signals. Figure 1b is the equivalent model of the system evaluated herein and shown in Fig. 1a. The equivalent model comprises a 420 V source that is connected to a 150 VA load based on the transmission line of the π-network.

**Figure 2:**
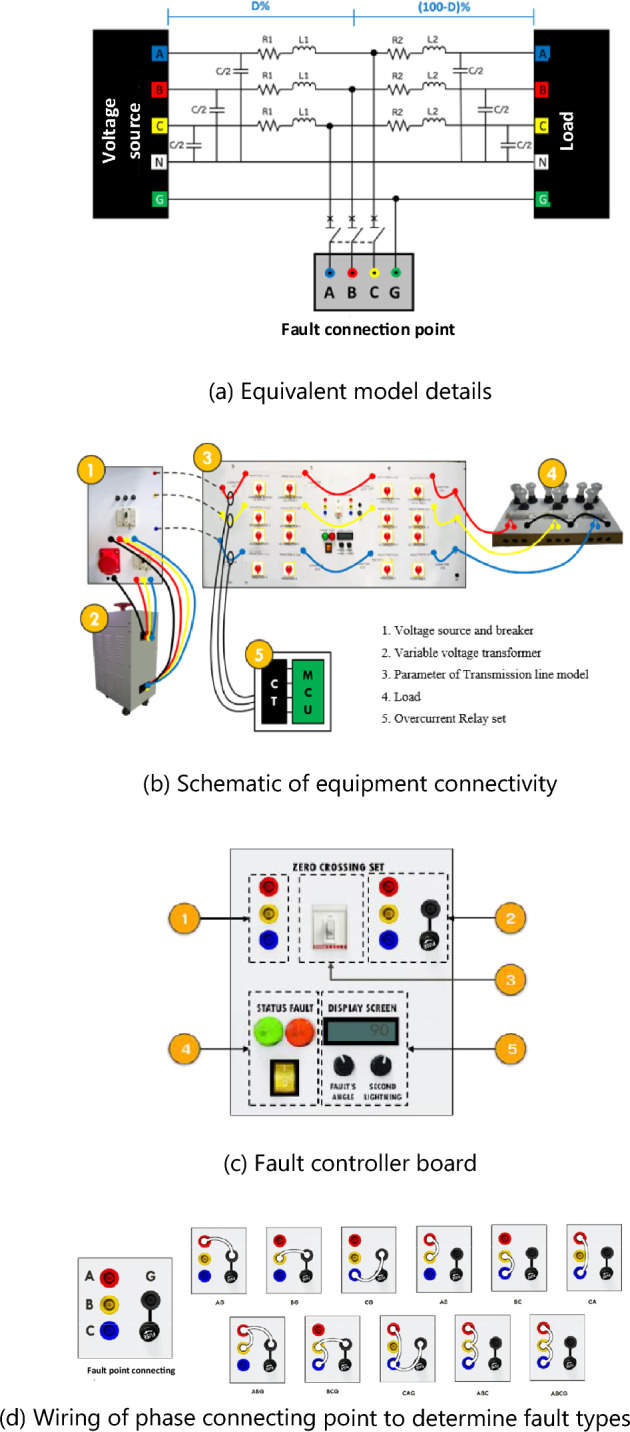
Experimental system.

The interconnecting components of the equivalent model are shown in Fig. 2. The model shown in Fig. 2a comprises two sets of pi connections. The fault connection point is a fault occurrence point at various transmission line distances within 0–70%. The fault type can also be adjusted. The equipment connections are shown in Fig. 2b. Five sections of equipment were connected as follows:

1. A voltage source and breaker
2. A three-phase variable voltage transformer (0–420 V)
3. Parameters of the transmission-line model
4. Load-containing lamps and ballasts
5. An overcurrent relay set.

However, when a fault occurs, the key point in the fault setting is the fault-controller board, as shown in Fig. 2c. The controller board consists of five sections:

1. The point that connects when a fault occurs
2. Phase connecting point
3. Switch on–off of the fault event
4. Fault-start switch
5. Voltage controller when a fault occurs.

After the experimental model was set, as shown in Fig. 2a–c, the type of fault was investigated by wiring a phase-connecting point, as displayed in Fig. 2d. Eleven fault types (AG, BG, CG, AB, AC, BC, ABG, ACG, BCG, ABC, and ABCG) were investigated.

After the experimental system was set up, the hardware used in this study was explained. The hardware consists of two parts: a Microcontroller unit (MCU) and a current transformer.

### Microcontroller unit: MCU

**Figure 3:**
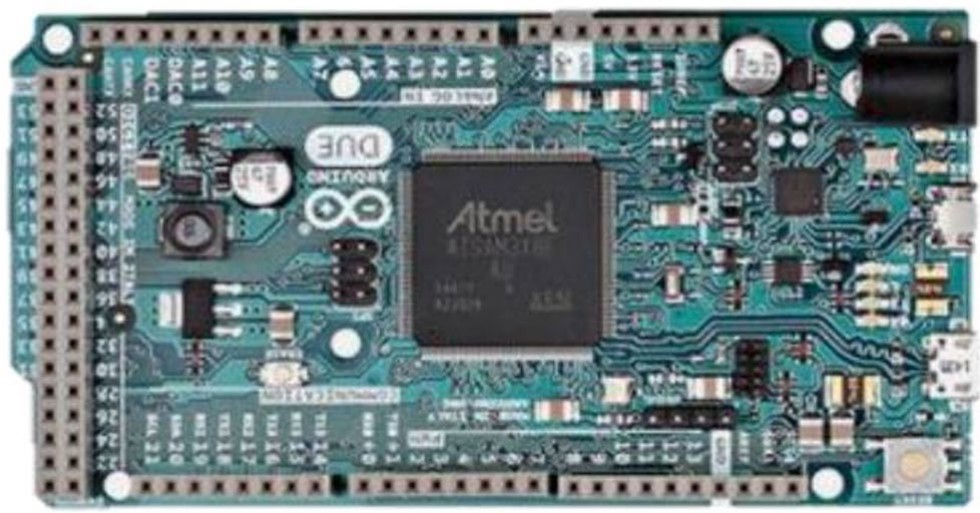
The Arduino DUE.

The MCU used in this study was an Arduino DUE with an 84 MHz clock, including an ARM 32-bit. The technical features of the Arduino DUE are shown in Fig. 3 and Table 2.

**Table 2:** Microcontroller Specification.

Microcontroller	AT91SAM3X8E
Voltage	3.3 V
Input Voltage (recommended)	7–12 V
Input Voltage (limits)	6–16 V
Digital I/O Pins	54 (of which 12 provide PWM output)
Analog Input Pins	12
Analog Output Pins	2 (DAC)
DC Output Current on all I/O lines	130 mA
DC Current for 3.3 V Pin	800 mA
DC Current for 5 V Pin	800 mA
Flash Memory	512 KB all available for the user applications
SRAM	96 KB (two banks: 64 KB and 32 KB)
Clock Speed	84 MHz

### Current transformer

**Figure 4:**
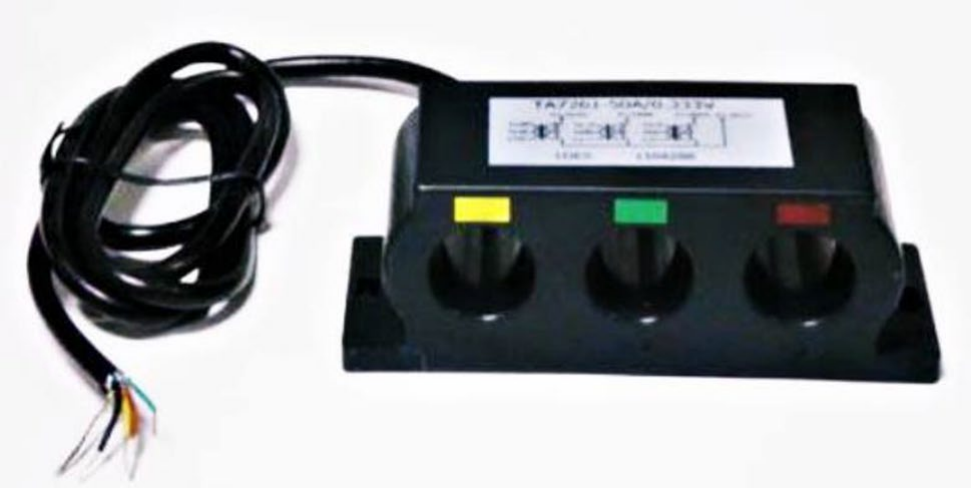
Three-phase current transformer, rated at 50 A/333 mV.

The maximum current of the current transformer (CT) used in this experiment was 7 A. Therefore, a 50 A rating was the closest we could find. The accuracy of the metre (accuracy class), which indicates measurement accuracy, was set to 0.2. This accuracy class is suitable for calibration because of an error value of 0.2% and a phase discrepancy from the result of the current transformer of only 10% at the rating. Therefore, a three-phase current transformer was selected for this experiment, rated at 50 A/333 mV in the frequency band. We used 50–400 Hz, Class 0.2 secondary winding, and 3000 turns, as shown in Fig. 4.

## Use of WT for fault detection and classification

**Figure 5:**
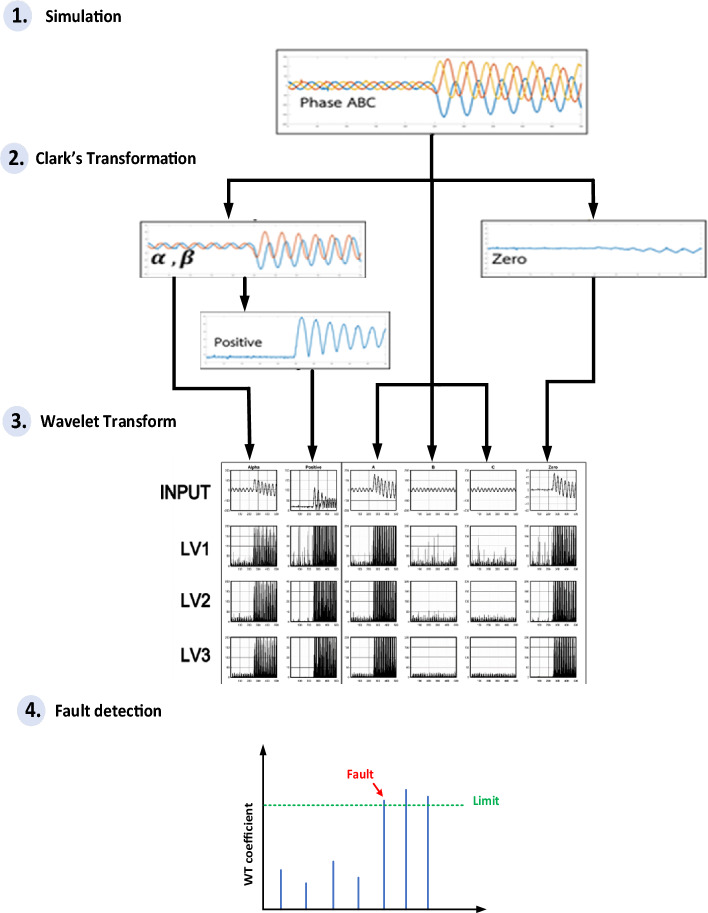
Flow chart process of overcurrent relay.

The signal analysis process (Fig. 5) used was as follows:

1. In the experimental model, the Arduino processor received a three-phase current signal from a current transformer with a voltage value of 0–333 mV. Analogue signals were then transformed into 12-bit digital values (0–4096).
2. Because the overcurrent relay has two operating functions (detection and classification faults), Clark’s transformation was applied to transform the current signal into phases A, B, and C. The input signals were simplified using Clark’s transform. The output signal is presented in order, with alpha (α), beta (β), and zero sequences. A positive sequence was integrated between the alpha (α) and beta (β) sequences. These four factors—phases A, B, and C, alpha sequences (α), positive sequences, and zero sequences—were used as the initial variables.
3. Third, a wavelet transform was applied to transform the initial variable signal. The output of this transformation can be used to analyse faults more clearly.
4. The current signal was handled using Clark’s and DWT transform.

The fault signal analysis process in this study has two objectives: fault classification and fault status identification. Therefore, the fault was detected using five system parameters (data for phases A, B, and C, and alpha and zero sequences). Phase and alpha sequence signal data were used to detect faults. A zero-sequence signal was used to classify the ground faults. Moreover, the data from the positive sequences were used for fault detection. The fault detection performance was compared using alpha and positive sequences. The output of the proposed algorithm provides a notification of system status. If a fault status is detected, the fault phase is annotated using the data from phases A, B, and C. On the contrary, when the system operates normally, the algorithm only displays the status.

### Clark’s transform

Clark’s transformation is a mathematical transformation employed to simplify the analysis of a three-phase circuit with its reform to alpha, beta, and zero sequences. The three-phase current can be reformed into three sequences using the following equations:

1 $$I_{{{{\upalpha \upbeta }}0}} (t) = \frac{2}{3}\left[ {\begin{array}{*{20}c} 1 & { - \frac{1}{2}} & { - \frac{1}{2}} \\ 0 & {\frac{\sqrt 3 }{2}} & { - \frac{\sqrt 3 }{2}} \\ \frac{1}{2} & \frac{1}{2} & \frac{1}{2} \\ \end{array} } \right]\left[ {\begin{array}{*{20}c} {I_{a} \left( t \right)} \\ {I_{b} \left( t \right)} \\ {I_{c} \left( t \right)} \\ \end{array} } \right]$$

where ${I}_{\alpha }$ = Alpha current sequence, ${I}_{\upbeta }$ = Beta current sequenssssssce, ${I}_{0}$ = Zero current sequence.

**Figure 6:**
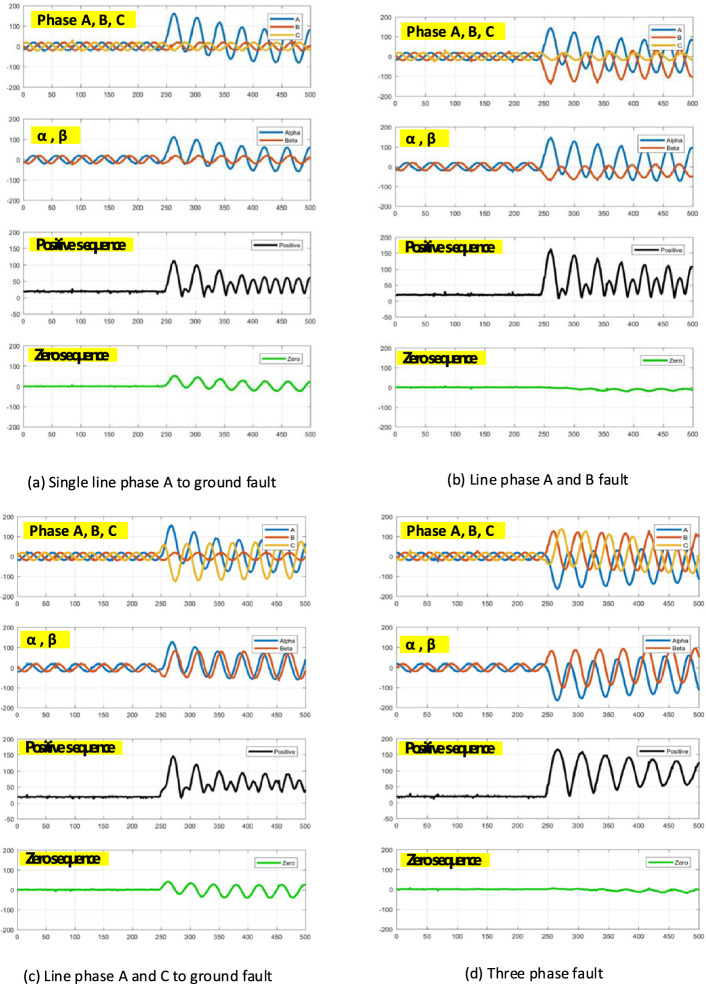
Clark’s transform.

Clark’s transform was applied in this study to transform current phases A, B, and C, recorded from the simulation, into the sequences of alpha (*I*α), beta (*I*β), and zero (*I*_0_). Four fault situations are displayed in Fig. 6. The characteristic of a single-line phase A fault to ground fault is displayed in Fig. 6a. Phase A and B faults are displayed in Fig. 6b. Comparing the signal between Fig. 6a,b, when a fault occurs, only the current of the fault phase increases. The results show that the amplitude of alpha, beta, and the positive sequence increases too. However, the amplitude of the zero sequence increases in cases of fault to ground. Moreover, considering the fault characteristic displayed in Fig. 6c,d, it displayed a similar behaviour. Therefore, it can be concluded that the current phase increases with the fault occurrence. Meanwhile, the zero sequence increases with the ground fault. In addition, the alpha and beta sequences increased as the fault phase current increased. The results of the positive sequence, which combined the alpha and beta sequences, followed the same trend.

**Figure 7:**
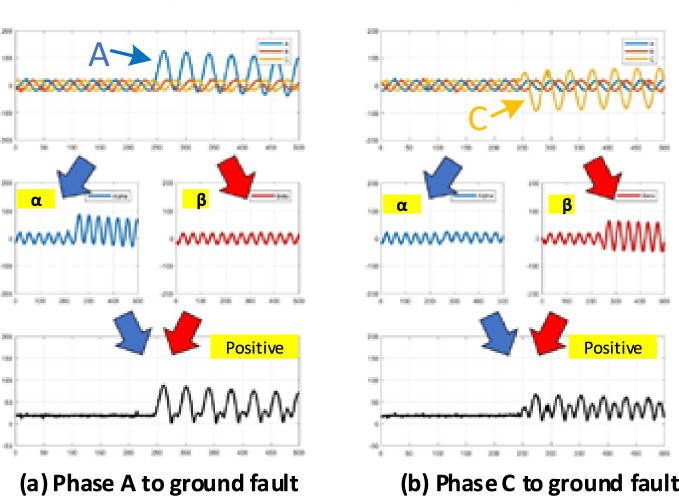
Fault characteristics based on Clark’s transform.

However, when considering the characteristics of a single-line phase A to ground fault and phase C to ground, as displayed in Fig. 7a,b, the following interesting findings were obtained: First, we considered a phase A to ground fault. The current in phase A increases when the fault phase suddenly increases. However, when considering the output of Clark’s transformation, only the alpha sequence showed an increase. The data sequence is maintained at this value.

However, when considering phase C to ground fault, even when phase C (fault phase) increased, only the beta sequence increased. The alpha sequence was unchanged. The reason for this change was that Clark’s transformation required a phase reference; thus, it referred to phase A. Therefore, the ratio of the phase was one time higher than that of the other phases (ratio phase A:B:C = 2:1:1). Therefore, faults occur in phases B and C. Fault detection based on Clark’s transform is erroneously detected or not detected. It efficiently detected faults only when they occurred in Phase A.

To address this limitation, the positive sequence parameter has been proposed for use in fault detection. The positive sequences displayed in Fig. 7a,b show that even when the characteristics of alpha and beta were different, the positive sequences in both cases were the same, which increased with fault occurrence.

Concluding with all the characteristic observations, the data of phases A, B, C, and alpha sequences were set as the initial variables for fault analysis. Moreover, a positive-sequence factor was added as an alternative method for fault detection.

### Wavelet transform

The stipulation of the signal investigation was set. The Daubechies scale 2 (db2) family wavelet was used to determine the mother wavelet because the waveform was asymmetric on the left and right sides (similar to a fault signal). Simultaneously, the sampling rate was set to the maximum value the hardware could record. This maximum value was based on the single-cycle analysis duration of analogue-to-digital converter. Thus, the maximum sampling rate must be greater than the total duration of the analysis. Therefore, the wavelet transform was set at levels 1–3, and the sampling time was set to 0.5 s.

The experimental model shown in Fig. 2 was used, and current signals were recorded subject to the condition that the observed parameters varied.

1. Fault type: Four types of expected transmission system faults were observed; these included the SLG, LL, DLG, and 3P faults.
2. Fault phase: The fault phase varied (A, B, and C).
3. Fault distance: The distance of the transmission line from the fault position to the source was varied (40%, 50%, 60%, and 70%).

**Figure 8:**
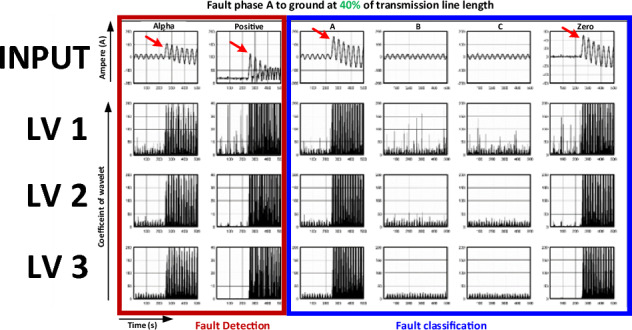
Current signals recorded in the case of single-line-to-ground (SLG) fault (phase A) at 40% of the transmission line length.

The current signal was recorded from the experimental model when the faults occurred. The signal is shown in Fig. 8 and is characteristic when an SLG fault (phase A to ground) occurs, and the location is 40% of the transmission line length. The characteristics of the alpha- and positive-sequence currents were determined from the experimental model using Clark transforms. The x-axis denotes the time (s), and the y-axis denotes the current (ampere (A)). Positive-sequence currents were analysed for fault detection. Simultaneously, the current phases A, B, C, and the zero sequence were analysed for fault classification. These characteristics were transformed using DWT to extract a coefficient wavelet. Three DWT levels are shown.

Considering the DWT signal levels 1–3 displayed in Fig. 8 (where the x-axis denotes time and the y-axis denotes the wavelet coefficient), it was found that the wavelet signal at level 1 had more noise than that at level 3. This is because faults occur in the high-frequency range. Thus, a high DWT level filters the high-frequency passes. Thus, low-frequency noise in the original signal was eliminated. For the same reason, the wavelet coefficients of the three-phase currents at level 3 were also more evident than those at levels 1 and 2.

**Figure 9:**
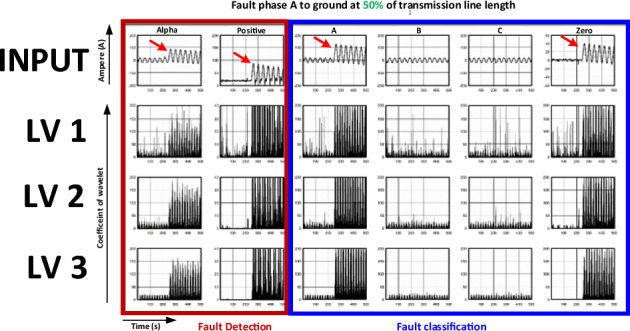
Current signals recorded in the case of the SLG fault (phase) A at 50% of the transmission line length.

In addition, the results relevant to the fault position change from 40 to 50% of the transmission line length are shown in Fig. 9. The magnitudes of the alpha- and positive-sequence currents were similar to the responses obtained when the fault occurred at 40% of the length of the transmission line. The magnitudes of both signals suddenly increased when the faults occurred. However, when the magnitude values were compared, the current magnitude in the 50% transmission line length case was lower than that in the case where the fault occurred at 40% of the transmission line length.

When considering the wavelet coefficients extracted at levels 1–3, it can be observed that the wavelet coefficient extracted at level 3 in the DWT case was the same as that obtained in the case of a 40% fault position. This is because the noise was eliminated by the DWT filter.

**Figure 10:**
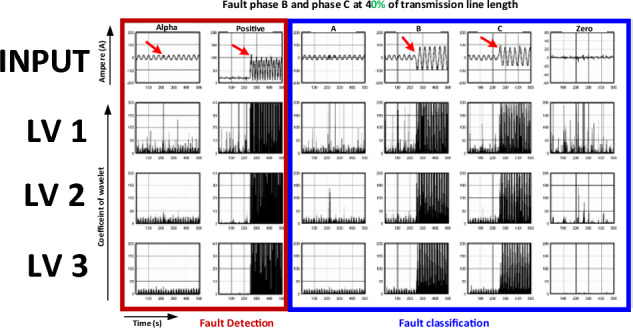
Current signals recorded in the case of line-to-line (LL) fault (phases B and C) at 40% of the transmission line length.

When the distance of the fault location was varied and the phase and type of fault were fixed, the variation in the distance directly affected the magnitude of the current. This is because the fault location (% distance) is related to the impedance. Thus, the current varies following Ohm’s law. Furthermore, the outcome when the phase and type of fault varied and the % distance of the transmission line was fixed is shown in Fig. 10.

The characteristics of the current displayed in Fig. 10 show three issues. First, only the magnitude of the positive-sequence current increases. The magnitude of the alpha sequence remains unchanged even when faults occur. This demonstrates that the alpha phase is unsuitable for fault analysis. A positive sequence is more suitable for fault analysis.

When the wavelet coefficient displayed in rows 2–4 of Fig. 10 is considered, the results have similar characteristics to those observed in the previous case, in which the coefficient extracted by the DWT (level 3) was more evident than the other two levels.

Considering the results in Figs. 8, 9 and 10, it can be concluded that the positive sequence was suitable for fault analyses because it included three-phase current data, and the phase ratio of A:B:C was 1:1:1. The alpha phase was not appropriate for fault analysis because the ratio of the alpha transformation was not a three-phase balance with a ratio of 2:1:1. Correspondingly, phase A remains unchanged even if faults occur in phases B and C. In addition, the level-3 DWT extracted the most evident wavelet coefficients.

**Figure 11:**
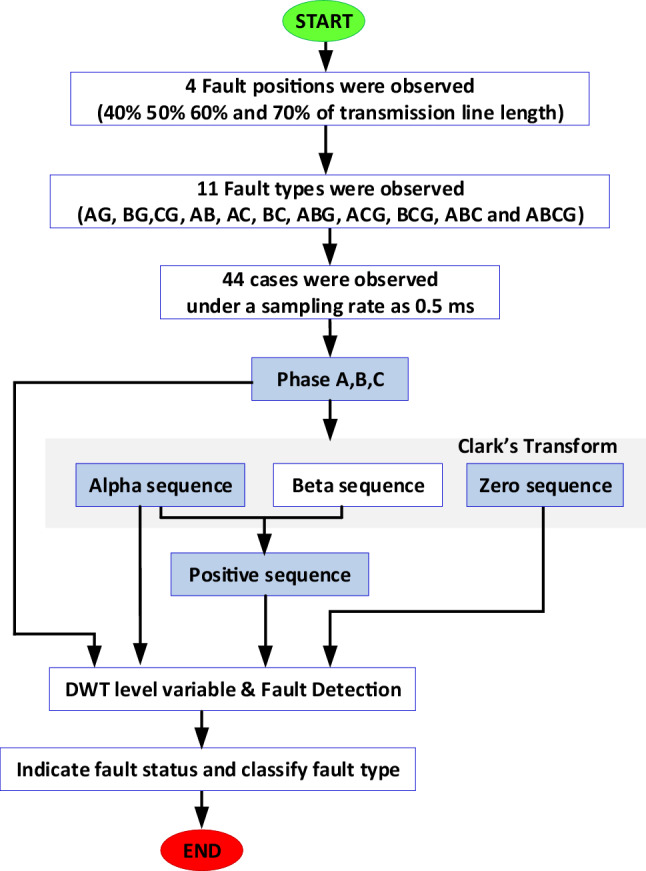
Fault analysis flow chart.

A flowchart of the fault analysis is shown in Fig. 11. First, the experimental model was based on the use of a 420 V source with a connected 150 VA load. A fault occurred at the transmission line, which linked the voltage source and load. The fault location varied from 40 to 70% of the transmission line, using the voltage source as the reference. Four types of faults (44 cases) were recorded, in which the current signals have 0.5 sampling times. Clark’s transform was used for the signal analyses. Six data points were used to determine the fault status and classify faults using the DWT, including the current phases: A, B, C, alpha sequence current, positive sequence current, and (6) zero sequence current. The fault detection performances based on the alpha and positive sequence currents were also compared.

## Discussion and analysis

The six-parameter data used for the fault analyses in this study are listed in Tables 3, 4 and 5. Fault analyses were divided into three procedures: fault detection, fault phase classification, and short circuit-to-ground identification.

**Table 3:** Time of fault recorded based on 0.5 sampling rate.

Alpha sequence current														
Method	% Position	SLG fault (ms)			LL fault (ms)			DLG fault (ms)			Three-phase fault (ms)		Total	
		AG	BG	CG	AB	AC	BC	ABG	ACG	BCG	ABC	ABCG	AVG time (s)	Case error
DWT Level 1	**40**	2	Fail	Fail	1.5	Fail	Fail	Fail	Fail	Fail	Fail	Fail	4.88	28
	**50**	Fail	5.5	Inf	1.5	Fail	Fail	Fail	4.5	1	4.5	Fail		
	**60**	Fail	Fail	Fail	2.5	12	Fail	Fail	5.5	Fail	2.5	4.5		
	**70**	Fail	15	Fail	Inf	Fail	Inf	3.5	Fail	Fail	5.5	6.5		
DWT Level 2	**40**	2	2.5	Fail	1.5	Fail	Fail	2	Fail	Fail	3	Fail	3.16	16
	**50**	1.5	3	Inf	1.5	Fail	Fail	2.5	5	2.5	3.5	Fail		
	**60**	3	3	Inf	2.5	1	Inf	2	6.5	7	3	4		
	**70**	2.5	3.5	Inf	2.5	Fail	Inf	2.5	5	Fail	5	5		
DWT Level 3	**40**	4	5	Inf	3.5	7	Inf	4	5.5	7	4.5	5	5.76	10
	**50**	4	6	Inf	4	3.5	Inf	4.5	7	9	5.5	5		
	**60**	5	6	Inf	5	4	Inf	4.5	8	Fail	6	6		
	**70**	5	16	Inf	5	3.5	Inf	5.5	8	Inf	7	7.5		

Positive sequence current														
Method	% Position	SLG Fault (ms)			LL Fault (ms)			DLG Fault (ms)			Three-phase fault (ms)		Total	
		AG	BG	CG	AB	AC	BC	ABG	ACG	BCG	ABC	ABCG	AVG time (s)	Case error
DWT Level 1	**40**	Fail	Fail	Fail	Fail	Fail	Fail	Fail	Fail	Fail	Fail	Fail	0.5	43
	**50**	Fail	Fail	Fail	Fail	Fail	Fail	Fail	Fail	Fail	Fail	Fail		
	**60**	Fail	Fail	Fail	Fail	Fail	Fail	Fail	Fail	Fail	Fail	Fail		
	**70**	Fail	Fail	Fail	Fail	Fail	0.5	Fail	Fail	Fail	Fail	Fail		
DWT Level 2	**40**	3	Fail	Fail	1.5	Fail	1.5	1	Fail	Fail	Fail	1.5	2	17
	**50**	3	2	2.5	1.5	Fail	1	1.5	Fail	1.5	1.5	Fail		
	**60**	3.5	2	2.5	2.5	Fail	Fail	1.5	Fail	1	1.5	1		
	**70**	3	2.5	Fail	Fail	Fail	1.5	3	3	Fail	2.5	Fail		
DWT Level 3	**40**	2.5	1.5	2.5	2	Fail	2	1.5	2.5	1	1.5	1.5	2.55	1
	**50**	3	3.5	4.5	1.5	2.5	1.5	2	4	2	1.5	1.5		
	**60**	4	2	4.5	3	2	1.5	2	4.5	1.5	1.5	1.5		
	**70**	4.5	4.5	6	3	2.5	1.5	3	4.5	0.5	2.5	3

**Table 4:** Classification of the fault phase when DWT and % of fault position varies.

Fault type		DWT Level 2				DWT Level 3			
		% 40	% 50	% 60	% 70	% 40	% 50	% 60	% 70
SLG	**AG**	False	False	True	True	True	True	True	True
	**BG**	Inf	True	True	True	True	True	True	True
	**CG**	Inf	True	True	Inf	True	True	True	True
LL	**AB**	True	True	True	Inf	True	True	True	True
	**AC**	Inf	Inf	Inf	Inf	Inf	True	True	True
	**BC**	False	True	Inf	True	True	True	True	True
DLG	**ABG**	False	False	True	True	True	True	True	True
	**ACG**	Inf	Inf	Inf	True	True	True	True	True
	**BCG**	Inf	True	True	Inf	True	True	True	True
Three-phase	**ABC**	Inf	True	True	True	True	True	True	True
	**ABCG**	False	Inf	False	Inf	False	False	False	False

**Table 5:** Result of fault detection and classification based on A 0.5 sampling rate.

% Accuracy					Time recorded (s)				
	DWT level 1	DWT level 2	DWT level 3	STFT		DWT level 1	DWT level 2	DWT level 3	STFT
Alpha sequence	36.36	63.64	77.27	81.82	Alpha sequence	4.88	3.16	5.76	5.17
Positive sequence	2.27	61.36	97.73	100	Positive sequence	0.5	2	2.55	5.16

The data in the table display the fault detection based on the alpha sequence current and positive sequence. Determine ‘Fail’ in the table is an error-detecting fault, and ‘Inf’ means cannot detect fault.

When considering fault detection based on the DWT of the alpha-sequence current, it can be observed that the level of the DWT directly influences fault detection. The fault detection case based on DWT level 1 exhibited the highest error detection when compared with the results of the fault detection at DWT levels 2 and 3. Simultaneously, even though DWT level 3 has the lowest error detection, several detection cases displayed an ‘Inf’ status or the fault cannot be detected. Moreover, the fault detection time based on DWT level 3 was longer than that of the other methods, although the detection performance was better.

Next, when considering fault detection based on positive sequence current, it can be seen that many cases detected errors when using DWT levels 1 and 2, while fault detection based on DWT level 3 was able to detect faults without ‘Inf’ status or ‘fault cannot be detected’ and fewer error detection cases (‘Fail status’). This is because the frequency range of DWT level 3 is approximately equal to the frequency range when a fault occurs.

We focused on the fault detection performance by comparing the data between the alpha and positive sequence currents. Thus, it can be concluded that detecting faults using a positive-sequence current is more efficient than using an alpha-sequence current. This is because the reference was set to phase A. When a fault occurred in phases B and C, the ratio of the phase was one time higher than that of the other phases (phase A:B:C = 2:1:1). Therefore, fault detection based on the additional Clark transform using alpha, beta, and positive sequence current was erroneously detected or not detected. It efficiently detected faults only when they occurred in phase A.

However, detecting faults using a positive-sequence current instead of an alpha-sequence current can overcome this drawback. The reason for this is to combine the alpha sequence with the beta sequence, as shown in the following equation:

2 $${I}_{+}=\sqrt{{({I}_{{\upalpha }})}^{2}+{({I}_{\upbeta })}^{2}}$$

where ${I}_{+}$ = Positive sequence, ${I}_{{\upalpha }}$ = Alpha current sequence, ${I}_{\upbeta }$ = Beta current sequence.

In addition, the detection time when using a positive-sequence current was shorter than when using an alpha sequence. Therefore, it can be concluded that fault detection based on the positive-sequence current and DWT filter at level 3 was efficient for fault detection in this study. The performance of the positive-sequence current based on DWT level 3 has only one case of error detection (total of 44 cases), and the time detection was 2.55 s.

After the fault was successfully detected, fault phase classification was considered. A positive-sequence current is efficient for fault detection. Therefore, in the identification phase, which is a continuation of fault detection, the positive-sequence data are considered, while the alpha-sequence current is disclaimed. The data in Table 2 are the results of fault classification at the DWT level and % of the fault position variable.

The data in Table 2 shows that in classifying faults using DWT level 2, there were many cases where the fault phase was false to detect (‘False’ status) and could not be detected (‘Inf’ status). Meanwhile, DWT level 3 was efficient in fault detection and fault classification. This was because of frequency, as mentioned previously. However, the classification of the fault phases using DWT Level 3 has some deficiencies in the case of three-phase and three-phase-to-ground faults. Because the grounding fault influences the amplitude of the current phase, the wavelet coefficient is also influenced. To solve this deficiency, the zero-sequence current parameter is applied to identify phase faults from short circuits to ground.

Finally, the results of the fault detection and classification are presented in Table 5. The time detection performance and % accuracy was considered.

First, we considered the % accuracy when using the alpha sequence current and the positive sequence current to detect and classify faults. Therefore, it can be concluded that a positive-sequence current is more efficient for fault detection and classification. Moreover, the level of the DWT is a significant factor that should be considered. The DWT level 1 results are less accurate, whereas the level of DWT increases the % accuracy.

Second, when considering time detection, the results showed that the fault can be quickly detected using a positive sequence with a time detection of less than 3 s. Meanwhile, time detection using an alpha sequence current was approximately 6 s.

Moreover, the result of DWT was compared to traditional method, Short-time Fourier transform (STFT), to verify the performance and reliability. The % accuracy was considering first, it can be seen that the accuracy of DWT method based on positive sequence was higher than the DWT method that based on alpha sequence. In which, the accuracy is approximately 97%. While, the accuracy when using STFT method is 100%. On the other hand, it found that time recorded when using STFT was longer that DWT which the time was 5.16 s and 2.55 s, respectively. Although the % accuracy of STFT was slightly higher than DWT but the time recorded is one times slower. Therefore, when analyzing the overall data in Table 5, the % accuracy was slightly different which it was insignificantly. However, the time is completely different so choosing DWT is more appropriate than STFT.

## Conclusion

In this study, a prototype protection relay was designed. The prototype was based on an actual 115 kV transmission system, which consisted of a 115 kV substation that transferred power to a 100 MVA load. The equivalent circuit of the experimental model comprised a 420 V voltage source that transferred power to a 150 VA load with a transmission line. Fault characteristics were observed under conditions in which the fault phase, type, and position varied. Clark’s transform and DWT were used for the analysis. The results showed that the proposed method, which is based on five significant factors, is efficient for fault detection and classification with acceptable fault detection times. A positive-sequence current signal is used for fault detection. Three current phases and zero-sequence current signals were used for the fault classification phase and short circuit to the ground. However, the DWT scale factor affects the detection accuracy. Therefore, optimal settings are essential for signal analysis. The findings of this research are beneficial to the development of electrical system protection devices. Reducing the limitation of existing protective device in term of accuracy and fast detecting and locating the fault which it will increase the reliability and stability of power system. Due to this paper found some obstacles which was limitation of microcontroller and noise effect. Therefore, future study, we suggest to (1) use more high quality of microcontroller which its time processing was faster and (2) use other equipment instead that has no effect of noise such as solid-state relay (SSR).

## Data Availability

The datasets used and analysed during the current study available from the corresponding author on reasonable request.
